# Protocol for whole-mount staining of melanized insect tissues for RNA and protein detection

**DOI:** 10.1016/j.xpro.2026.104921

**Published:** 2026-11-09

**Authors:** P. Daniel H. Pastor, Giacomo L. Glotzer, Dominic D. Frank, Ivan Lacroix, Alejandra Hurtado-Giraldo, Daniel J.C. Kronauer

**Affiliations:** 1 Tri-Institutional MD–PhD Program, Weill Cornell/Rockefeller/Sloan Kettering, New York, NY 10065, USA; 2 Laboratory of Social Evolution and Behavior, The Rockefeller University, New York, NY 10065, USA; 3 Howard Hughes Medical Institute, New York, NY 10065, USA

**Keywords:** Cell Biology, Microscopy, Molecular Biology, Neuroscience

## Abstract

Technical barriers have limited whole-mount staining and imaging of internal tissues in many insects and other arthropods. Here, we present a protocol for whole-mount staining of melanized insect tissues for RNA and protein detection. We describe steps for preparing, permeabilizing, and bleaching samples; designing RNA probes; and staining. We then detail procedures for imaging samples. This protocol is compatible with both immunohistochemistry and fluorescence *in situ* hybridization and enables high-resolution microscopy of tissues surrounded by cuticle without sectioning.

For complete details on the use and execution of this protocol, please refer to Glotzer et al.^1^

## Highlights

• Procedures for the permeabilization and bleaching of cuticularized insect tissues
• Steps for RNA and protein detection and imaging in cuticularized insect tissues
• Guidance on custom probe design for hybridization chain reaction (HCR)

## Abstract (editor highlights)

Publisher’s note: Undertaking any experimental protocol requires adherence to local institutional guidelines for laboratory safety and ethics.

## Graphical abstract

**Figure:**
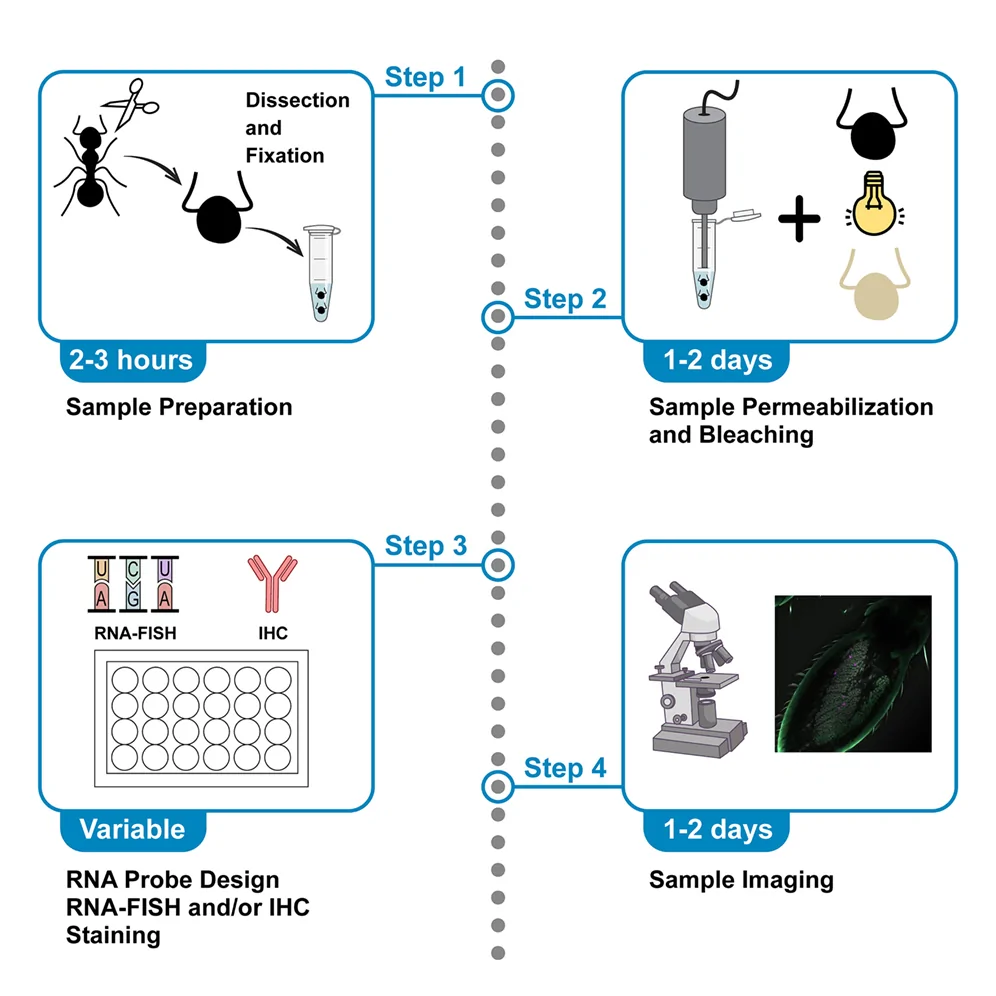

## Before you begin

This protocol describes whole-mount immunohistochemistry (IHC) and RNA fluorescence in situ hybridization (RNA-FISH) using HCR v3.0 reagents from Molecular Instruments.^2^ The technique preserves tissue integrity while enabling visualization of RNA and proteins in tissues that are encased in thick and melanized cuticle. Here, we primarily describe application of this protocol to antennae of the clonal raider ant, *Ooceraea biroi*, with additional experiments showing its use in staining other tissues, such as legs and thorax. We also report successful application of the protocol to whole-mount RNA-FISH in antennae of the flour beetle, *Tribolium castaneum*.

Melanized cuticle presents significant challenges for imaging. It acts as a physical barrier that prevents reagent penetration, it decreases optical accessibility at shorter wavelengths, and it increases the requirement for high laser power during imaging. Because the cuticle hardens and darkens as arthropods age, it especially limits imaging feasibility in older individuals.^3^^,^^4^^,^^5^^,^^6^ Our protocol overcomes these limitations through a combination of gentle sonication and peroxide methanol bleaching, followed by standard immunohistochemistry and HCR hybridization steps. Together, these treatments enhance tissue permeability and signal detection while maintaining tissue integrity and overall anatomy.

Although optimized for antennae of the clonal raider ant, the protocol can be adapted to other ant species and to other insects. It is particularly suitable for cuticular structures. Additionally, to enable flexible, genome guided probe design for multi species RNA-FISH experiments, we developed ProbePy, a Python package that streamlines the design, screening, and ordering of highly specific probes compatible with multiplexed HCR amplification. All reagent recipes are provided in the materials and equipment section. All steps related to RNA-FISH should be performed under RNase-free conditions.

### Preparation of transfer baskets for rapid well-to-well handling during FISH and IHC

We find that the traditional use of microcentrifuge tubes for RNA-FISH, which requires numerous washes and solvent changes, is cumbersome. Using movable baskets and well plates instead improves workflow efficiency and helps prevent tissue damage or loss. The baskets and well plates can be used at any step that normally uses traditional microcentrifuge tubes. The only difference is the need to maintain proper liquid levels in the well plates because the setup is more prone to evaporation. We find that for a 24-well plate the minimum is 350 μL, which is reasonable for IHC and FISH protocols. In this protocol, we start using the plates post-sonication as we find it easier to sonicate right after fixation with microcentrifuge tubes.

Before beginning, prepare the baskets (one for each sample/batch of samples) and 24-well plates using sterile, RNase-free tools (Figure 1). All tools are treated with RNase Displace™ prior to use. The number of samples and solvent changes will dictate the number of plates used.

**Figure 1:**
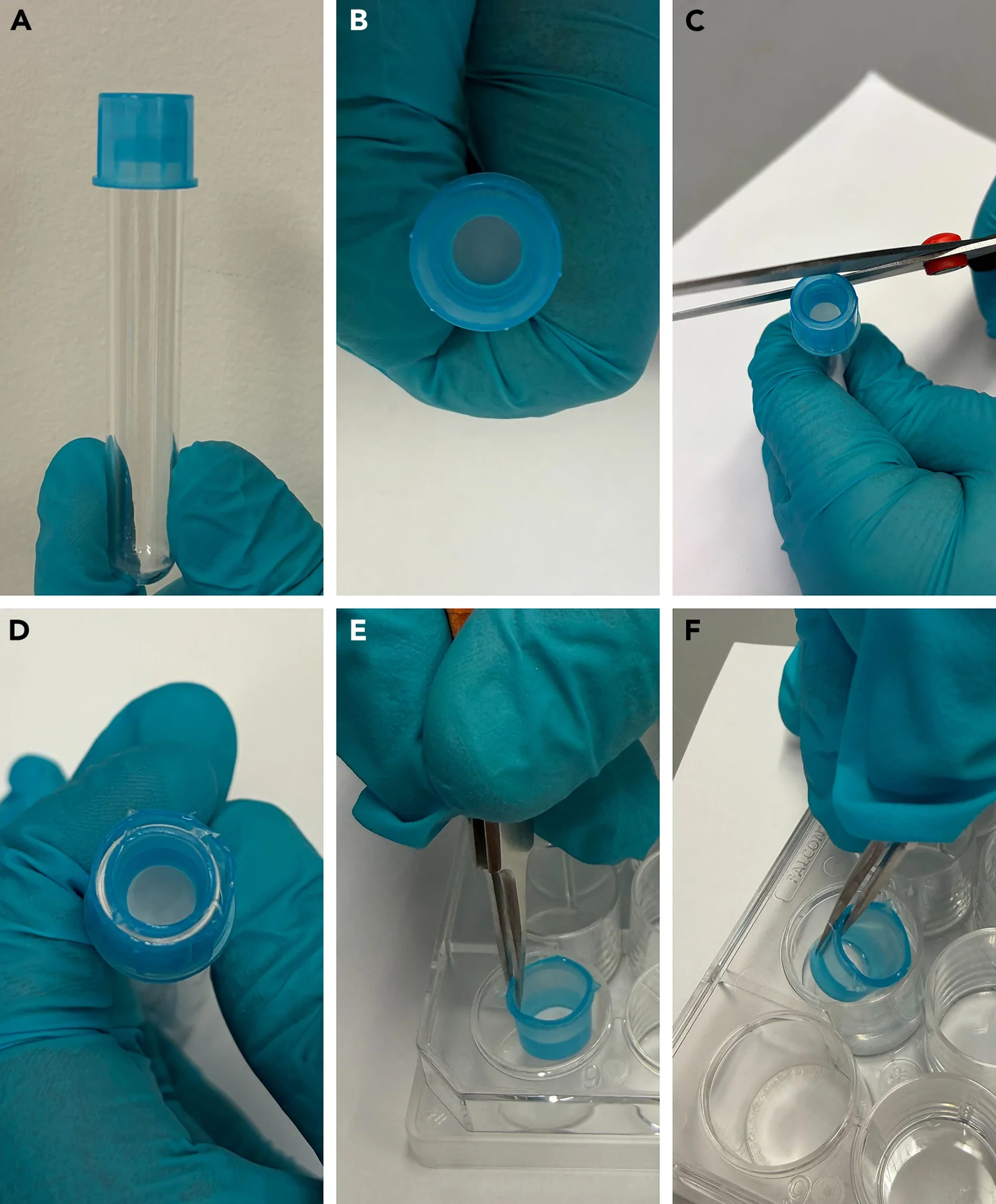
Preparation of the basket and well system for RNA-FISH staining (A) A 5 mL round-bottom polystyrene test tube with the internal basket intact prior to modification. (B) Top view of the test tube, showing the circular basket seated inside the tube. (C) With the cap left attached for stability, scissors are used to cut along the upper edge of the basket to separate it from the tube. (D) The basket after separation from the tube, shown from above. (E) The detached basket is transferred into a well of a 24-well plate using tweezers. (F) The basket can be positioned at a 45° angle and then one end can be placed into the solution for 10 seconds before gently lowering the remainder of the basket. This method is particularly effective to allow for proper submersion in viscous liquids.

For example, if working with four samples, a 24-well plate allows for six solvent changes/washes for each sample, while with twelve samples, each plate allows for two solvent changes/washes for each sample. In the four-sample example, the plate should be turned 90 degrees from usual such that there are 4 columns and 6 rows. Each sample will occupy a column, and the basket will move downward from one row to another in each solvent change. In the twelve-sample example, the plate is positioned normally (6 columns and 4 rows), and the twelve samples are positioned in the first twelve wells (A1-6 and B1-6) for a solvent change. For the second solvent change, the samples are moved to the last two rows (C1-6 and D1-6).

If you have upwards of twelve samples, we recommend splitting them up into several plates (e.g., sixteen samples can be split up into four plates with four samples each, where each plate allows for six solvent changes/washes per sample). We recommend calculating how many plates you will use before starting based on the number of samples and solvent changes/washes you have.

1. In a sterile, RNase-free environment, prepare one 5 mL round-bottom polystyrene test tube for each batch of samples. The test tube contains a cell strainer in the blue cap (referred to in this protocol as a basket once it has been cut off the cap), which we find works well for the 24-well plate.
2. With the cap still attached for stability, use scissors to cut along the top edge of the basket to separate the basket from the outer edge of the blue cap. Figure 1C shows the basket attached to the cap before cutting. The position of the scissors shows what edge to cut. Figure 1D shows the cut basket still inside the test tube cap.
3. Using a pair of tweezers, remove the basket from the cap and place it into a well of a 24-well plate.
4. Repeat Steps 2 and 3 to match the number of baskets to the number of samples you have.

### Design of probe sequences using ProbePy

To enable RNA-FISH in various species, we designed ProbePy, a Python package that facilitates transparent probe design given any annotated genome and transcriptome. The probes are compatible with the B-system amplifiers from Molecular Instruments, enabling multiplexing of up to 5 channels and rapid amplification. Across taxa, recently duplicated chemosensory genes are especially difficult to probe due to their high sequence similarity. ProbePy supports rapid screening of genes to assess their uniqueness for specific labeling. Even if a target gene is too similar to another gene, the user can elect to allow off-target binding to this gene. ProbePy additionally lets the user choose the transcript isoform to target, whether to label introns, and the number of probe pairs per gene. The output files can be directly uploaded to IDT oPools, enabling the user to begin staining within days. To help users get started, we have included several tutorials on GitHub for how to prepare the data and design custom probes.

### Innovation

This protocol facilitates RNA-FISH and immunohistochemistry in thick and melanized insect tissues that are notoriously difficult to label in whole-mount preparations. The protocol incorporates gentle sonication to enhance probe and antibody penetration, as well as peroxide bleaching to improve imaging of short wavelength fluorophores. This workflow enables high-throughput whole-mount staining and imaging of tissues that previously required labor intensive cryosectioning.

### Institutional permissions

This study did not require special approval for animal use. Researchers should obtain any necessary approvals in accordance with institutional and federal guidelines.

## Key resources table

**Table:** 

REAGENT or RESOURCE	SOURCE	IDENTIFIER
**Antibodies**		

Mouse monoclonal anti-Orco (1:250)	Gift from Vanessa Ruta; Butterwick et al.^7^	clone 20F7
Anti-Synapsin (1:20)	Developmental Studies Hybridoma Bank	DSHB: 3C11; RRID: AB_528479
Donkey anti-mouse Alexa 647 (1:500)	Invitrogen	A32787; RRID: AB_2762830

**Chemicals, peptides, and recombinant proteins**		

DAPI	Thermo	D1306
Tween© 20	Sigma-Aldrich	P9416
HCR probe hybridization buffer	Molecular Instruments	N/A
HCR probe wash buffer	Molecular Instruments	N/A
HCR amplification buffer	Molecular Instruments	N/A
Triton X-100	Sigma-Aldrich	93443
SlowFade™ Glass Antifade Mountant	Invitrogen	S36917
Zeiss Immersol-G immersion media	Carl Zeiss Microscopy	462959-9901-000
1× Phosphate-buffered saline (PBS) without Ca^2+^/Mg^2+^	Corning	21-040-CV
10× Phosphate-buffered saline (PBS) without Ca^2+^/Mg^2+^	Thermo Scientific	J63521-AP
20× Saline-Sodium Citrate (SSC)	Invitrogen	15557044
16% Paraformaldehyde (PFA)	Electron Microscopy Sciences	15710
Normal Donkey Serum	Abcam	AB7475
Methanol 100% (1L)	Fisher Scientific	A412-1
Hydrogen peroxide 30%	Millipore Sigma	1072090250

**Experimental models: Organisms/strains**		

*Ooceraea biroi* clonal line B wild type	Kronauer Lab	Female adults of unknown age
*Tribolium castaneum*	Carolina	144356; adults of unknown age or sex

**Oligonucleotides**		

OR 9E12 (*Ooceraea biroi*)	Custom design; IDT production	See Table S1
Orco (*Tribolium castaneum*)	Custom design; IDT production	See Table S2

**Software and algorithms**		

Fiji/ImageJ	Fiji	RRID: SCR_002285 (https://fiji.sc)
Python version 3.10.0	Python Software Foundation	https://www.python.org
ProbePy	This study	https://github.com/Social-Evolution-and-Behavior/ProbePy

**Other**		

Tubes, 0.2 mL, domed cap	Eppendorf	AB0337
Dumont Fine Tweezers	Dumont	0207-5TI-PO
Vannas-Tübingen Spring Scissors	Fine Science Tools	15008-08
Sylgard™ Dissection Dishes (Small and Large)	Living Systems Instrumentation	DD-50
Entomology Forceps	Bioquip	N/A (Not available anymore, but any broad forceps for handling insects will do)
Olympus SZ61 Stereoscope	Multiple Vendors	N/A
Schott ACE 1 Light Source	Multiple Vendors	SKU: A20500
RNase Displace™	Decon Labs	1401
Falcon 5 mL round bottom polystyrene test tube, with cell strainer snap cap	Corning	352235
Multiwell 24 well	Corning	353047
QSonica Q700	QSonica	Q700-110
Mixer HC (without block) or any 37°C shaking incubator or hybridization oven; see the note at Step 31	USA Scientific	8012-0000
Lab Jack “8 × 8” Anodized Blue	Thermo Fisher Scientific	14-673-52
Fisherbrand™ Cover Glasses: Circles (#1.5)	Fisher Scientific	2-545-81P
Fisherbrand™ Microscope Slides, with Frosted Tabs (25mm x 75mm x 1mm)	Fisher Scientific	22-034486
Nail Polish (Clear)	Electron Microscopy Sciences	72180
Avery® Self-Adhesive Hole Reinforcement Stickers, 1/4″ Diameter	Avery	5720
Zeiss LSM 900	Zeiss	N/A
IsoFreeze Micro-Tube Chillers, Purple/Pink Indicator, 1.5 to 2.0 mL Tubes, 24 Tube Capacity	RPI	248014

## Materials and equipment

Below are the reagent recipes for several steps in tissue preparation, RNA-FISH, and immunohistochemistry. All reagents should be prepared fresh on the day of use unless otherwise noted.

**Table:** 8% formaldehyde stock solution

Reagent	Final concentration	Amount
16% Formaldehyde	8%	10 mL
10% Triton X-100	0.1%	200 μL
10× PBS (without Ca2+/Mg2+)	1×	2 mL
RNAse-free H2O	N/A	7.8 mL
Total (8% formaldehyde stock)	N/A	20 mL

**CRITICAL:** Formaldehyde is toxic and carcinogenic. Prepare these solutions in a fume hood and use appropriate protective equipment at all times.

***Note:*** The 8% formaldehyde stock solution can be stored at −20°C for up to 8 months.

**Table:** 4% formaldehyde working solution

Reagent	Final concentration	Amount
8% formaldehyde with 0.1% Triton X-100	4%	500 μL
RNAse-free H2O	N/A	500 μL
Total (4% formaldehyde stock)	N/A	1 mL

***Note:*** The 4% formaldehyde working solution should be prepared fresh for each experiment.

**Table:** 5× SSCT solution

Reagent	Final concentration	Amount
20× SSC	5×	10 mL
10% Tween©-20	0.1%	400 μL
RNAse-free H2O	N/A	29.6 mL
Total (5× SSCT)	N/A	40 mL

***Note:*** The 5× SSCT solution should be prepared fresh. Any remaining solution may be kept at room temperature and used within 1 week.

**Table:** 1× PBST (Tween©-20)

Reagent	Final concentration	Amount
1× PBS (without Ca2+/Mg2+)	1×	49.5 mL
10% Tween©-20	0.1%	500 μL
Total	N/A	50 mL

***Note:*** The 1× PBST (Tween©-20) solution should be prepared fresh. Any remaining solution may be kept at room temperature and used within 1 week.

**Table:** Normal Donkey Serum blocking buffer

Reagent	Final concentration	Amount
Normal Donkey Serum	5%	500 μL
100% Triton X-100	0.5%	50 μL
1× PBS (without Ca2+/Mg2+)	1×	9450 μL
Total	N/A	10 mL

***Note:*** The Normal Donkey Serum blocking buffer solution should be prepared fresh. Any remaining solution may be kept at 4°C and used within 1 week.

**Table:** 1× PBST (Triton X-100)

Reagent	Final concentration	Amount
100% Triton X-100	1%	100 μL
1× PBS (without Ca2+/Mg2+)	1×	9900 μL
Total	N/A	10 mL

***Note:*** The 1× PBST (Triton X-100) solution should be prepared fresh. Any remaining solution may be kept at room temperature and used within 1 week.

## Step-by-step method details

### Dissection and fixation of ant antennae

**Timing: ∼3 h**

The sample preparation section describes dissection and fixation of insect cuticular tissues prior to sonication. The steps below focus on antennae but can be readily adapted to the head, thorax, abdomen, and legs. The protocol can be performed with any number of antennae; however, a minimum of 20 antennae is recommended to account for potential sample loss during sonication.

1. Prepare a clean dissection area by wiping down the materials and dissection surface with RNase Displace™.
2. Place a tube containing 1 mL of 4% formaldehyde and a separate tube with 5 mL of PBST (PBS + 0.1% Tween©-20) in an ice bucket.
3. Cold anesthetize the ant colony by placing the dish containing the ants on ice for 10 min until fully immobilized.

***Optional:*** Ants can also be anesthetized with CO_2_.

4. Transfer several ants at once into a 1.5 mL centrifuge tube with 95% EtOH using forceps and submerge them for 30–60 seconds.

***Note:*** Any forceps that do not damage the cuticle work. If forceps are unavailable, nudging several ants from their dish into the tube with a pipette tip or pin works as well.

***Note:*** The 95% EtOH is used to make it easier to submerge the ants in aqueous solutions. We believe, but have not confirmed, this helps break the surface tension created by the cuticular hydrocarbons on the surface of the ants.

5. Remove the ants that were washed in EtOH using entomology forceps.
  a. Gently and briefly blot them on tissue paper to remove excess EtOH.
  b. Transfer them into a Sylgard™ dish filled with PBST.

***Note:*** Make sure all the ants are submerged to prevent drying out and damaging the tissue.

***Note:*** The Tween©-20 in the PBST helps to keep the samples submerged.

6. While submerged in PBST, remove the antennae with microdissection scissors near the joint where the antennae attach to the head.

***Note:*** Antennae still attached to the head have been processed through this protocol and have been successfully stained and imaged. However, dissection of the antennae, if that is the tissue of interest, allows for more antennae to be sonicated simultaneously. We have not noticed a difference in efficiency based on where the antennae were dissected. The base just represents a reproducible landmark.

7. Quickly transfer the antennae after dissection to the 4% formaldehyde in a 1.5mL microcentrifuge tube on ice.

***Note:*** Fine dissection tweezers can be used to transfer the antennae individually after each is dissected without transferring much PBST. However, we find it easier to transfer them in batches after several dozen have been dissected with P20 pipettes and tips. The antennae do not stick to the wall of the pipette tips with Tween©-20 in the PBST.

***Note:*** We find that holding the pipette vertically for a few seconds after pipetting up all the loose antennae so that gravity pulls them down to the tip of the pipette allows one to transfer the antennae to the formaldehyde without excess PBST.

***Note:*** If processing them in batches, we recommend no more than 30 minutes for dissections per batch. Longer dissection times before transfers to formaldehyde can cause tissue damage to the first antennae dissected in the batch.

***Note:*** After 1 h, or two 30 min dissection periods, we recommend that the antennae in formaldehyde on ice be transferred to RT or the cold room. This is to prevent unequal fixation between the first and last antenna transferred to formaldehyde. A new tube of fixative can then be used for another round of dissections.

8. After all antennae have been dissected, fix the tissue in 4% formaldehyde by gently rocking on a nutator at 25–50 rpm for 1 h at RT or for 2 to 5 days at 4°C.

**Pause point:** Stop here if needed, skip the incubation at room temperature, and leave the samples in fixative for 2 to 5 days at 4°C.

### Permeabilization and bleaching of ant antennae

**Timing: ∼1 h hands-on; 8–48 h total time**

The following section is critical to permeabilize the cuticular tissue. The settings and operation described here are for the Q700 Sonicator (QSonica, Newtown, CT) fitted with a 1.6 mm microtip and processing *O. biroi* antennae. Empirical testing of sonication amplitude and time may be necessary for different tissues and other sonicator models. To troubleshoot settings for different sonicators and tissues, we recommend dissecting and fixing tissue as above, sonicate with different parameters (include a non-sonication control), stain with DAPI at 1:300 to 1:500 for at least 1 h, and image immediately to visualize the effectiveness of staining.

9. Spin down the samples in a tabletop microcentrifuge and carefully remove the 4% formaldehyde from the 1.5 mL microcentrifuge tube without disturbing the antennae that have settled at the bottom.
10. Wash the samples twice with 500 μL PBST for 5 min.
11. Place the samples in a final solution of 500 μL PBST for sonication.
12. Prepare the samples to be sonicated as in Figure 2:
  ***Note:*** First, position the sonicator tip at the desired depth relative to the samples and mark this position on the probe. Then place the tubes in the chilled microcentrifuge-tube rack for sonication. Marking the position beforehand is useful because the rack can obscure the view of the samples once the tubes are in place.
  a. Open the 1.5 mL centrifuge tube containing the samples.
  b. Lift the tube toward the sonicator tip and adjust its position until the tip is approximately 5 mm above the samples at the bottom.
    ***Note:*** The bottom of the tube should be close to the sonicator tip, but the tip should not touch the samples at the bottom of the tube.
  c. Once the desired distance is reached, mark the sonicator probe at the point corresponding to the top edge of the centrifuge tube using a permanent marker.
  d. Remove the cooled microcentrifuge tube ice rack from the freezer and place it on the jack.
  e. Place the 1.5 mL centrifuge tube containing the samples into the center well of the rack and align it so that the tip is centered over the tube.
  f. Use the jack to gently raise the rack until the top edge of the tube aligns with the marker on the sonicator tip.
  g. Make minor adjustments to center the tip by gently moving the rack.

**Figure 2:**
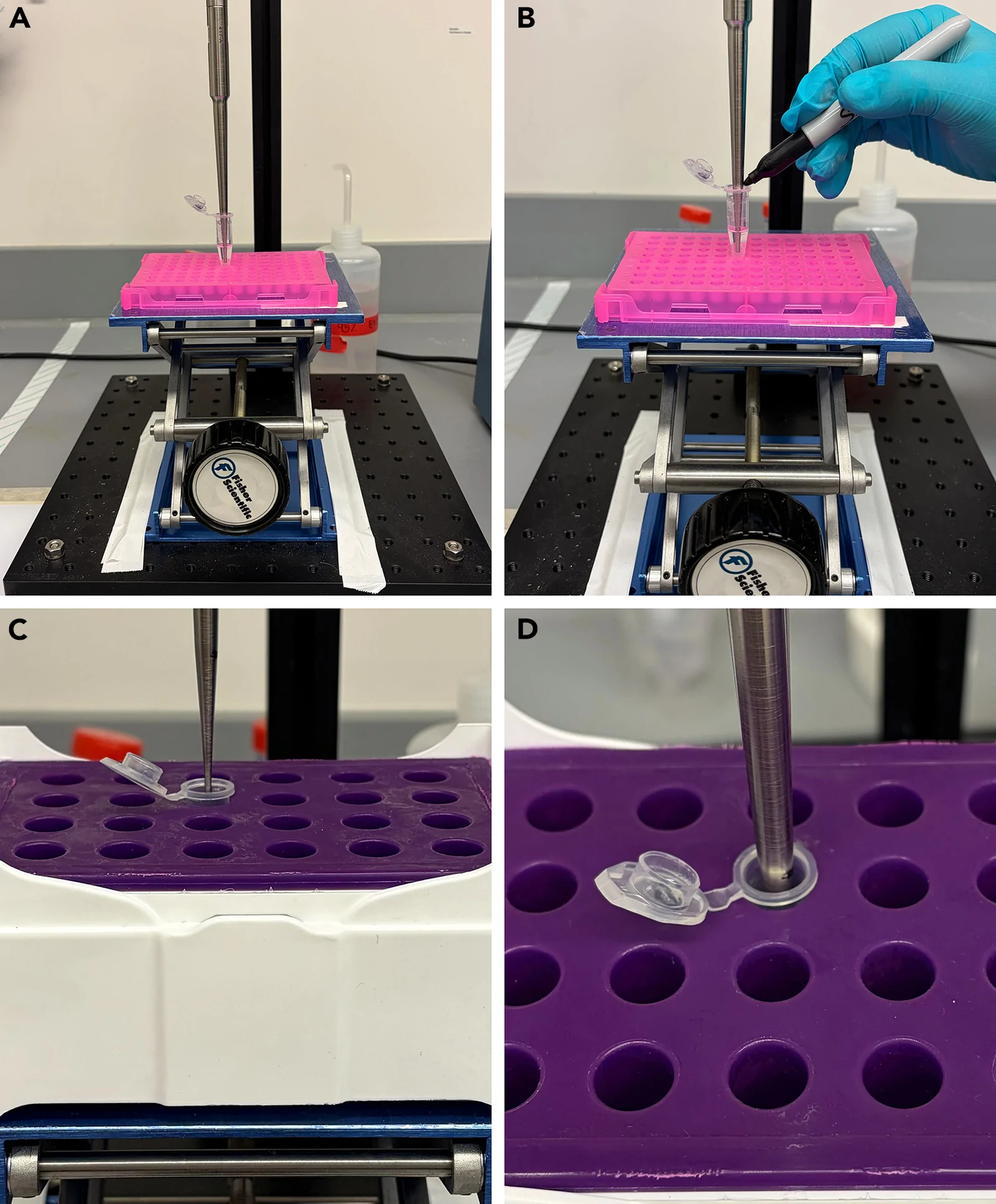
Sonication setup to permeabilize insect cuticle to stains, antibodies, and FISH probes (A) The image shows the sonicator probe tip, sample tube, and jack used to position the sample during sonication. (B) Using the jack, the tube is raised while held in place by a PCR tube rack until the sample is approximately 5 mm from the probe tip. A line is marked on the sonicator probe with a permanent marker at the level of the top edge of the tube. (C) The PCR tube rack is replaced with a pre-chilled IsoFreeze Micro-Tube Chiller, and the tube is placed into one of the wells. The probe tip is centered over the tube opening. (D) The jack is raised until the top edge of the tube again reaches the marked line, ensuring that the probe tip is positioned at the correct distance from the sample.
13. Prepare the sonicator:
  a. Turn on the sonicator.
  b. Menu options:
    i. Yes, using microtip.
    ii. Manual operation.

**Table:** 

	Mild	Normal
Amplitude	3	10
Pulse ON (seconds)	2	2
Pulse OFF (seconds)	20	20
Cycles	20	20

***Note:*** Amplitude may vary depending on the tissue. Softer tissues may benefit from a lower amplitude. In general, amplitudes between 3 (mild) and 10 (normal) have been effective. The shown settings are for the Q700 Sonicator when permeabilizing *O. biroi* antennae.

***Note:*** Total time reflects the total time the sonicator is ON. For the above settings, the display will indicate a total ON time of 40 s.

**CRITICAL:** Before running the sonicator, ensure adequate hearing protection is worn, and appropriate signage is posted to prevent entry into the room during operation.

14. Run the program until the sonicator has stopped, then lower the jack to remove the probe from the tube.
15. Clean the sonicator tip thoroughly, first with 95% ethanol and then with RNase-free water.
16. Spin down the samples that are in the microcentrifuge tube in a tabletop microcentrifuge and wash twice in PBST for 5 min, spinning between each wash.
17. Post-fix the samples in 4% formaldehyde by gently rocking on a nutator at 25 rpm for 1 h at RT.
18. Repeat Steps 12–17 for additional samples.

***Note:*** Multiple samples can be processed in parallel. While one sample is being sonicated, previously processed samples can be washed, fixed, and stored on ice until all samples are complete. If processing multiple samples, the cooled microcentrifuge tube rack will warm up and needs to be replaced with a new rack directly from the freezer. It is important to keep the samples cool while sonicating, because the procedure generates heat that can damage the tissue.

19. After all the samples have been post-fixed, spin down the samples and wash them twice in 500 μL PBST for 5 min. Samples may be held on ice or at 4°C until all batches have been processed before proceeding to Step 20.
20. Prepare a 24-well plate with one basket for each batch of samples, as described in the before you begin section.
21. Pipette up the antennae at the bottom of each microcentrifuge tube and transfer them to the center of a basket placed in a well containing 500 μL PBST.

***Note:*** We find that a P1000 pipette tip works well for transferring antennae. For larger specimens, we recommend cutting the end of the tip to create a wider opening.

22. Wash twice in 500 μL PBST for 5 min by transferring the basket to a new well containing fresh PBST every 5 min.
23. Place the 24-well plates on ice or in a cold room as all the dehydration steps should be done at 4°C.
24. By moving the basket to a new well each time, dehydrate the samples through a graded methanol/PBST series: 25%, 50%, 75%, and twice in 100%. Incubate each step on ice on a nutator at 25 rpm for 10 min.

***Note:*** A total of six wells per sample should be used here.

**Pause point:** Samples may be stored for up to 6 months at −20°C in 100% methanol. Long-term storage should be done in microcentrifuge tubes (to prevent evaporation) in flammable safe freezers.

25. Prepare a 3% H_2_O_2_/90% methanol solution by combining 100 μL of 30% H_2_O_2_ with 900 μL of 100% methanol per sample in a fume hood.
26. Move the baskets to a new well with 1 mL of the 3% H_2_O_2_/90% methanol solution.

**CRITICAL:** If bubbles emerge from the tissue upon addition of peroxide, dehydration was incomplete. Discard affected samples and repeat dehydration with fresh tissue, extending each step to 20 min.

27. Place 24-well plates containing samples and peroxide solution on a nutator in the cold room or on top of a bucket filled with ice placed on a nutator at 25 rpm.

***Note:*** Position a light source approximately 5 cm from the plate and illuminate at maximum power (Schott ACE 1 Light Source), as shown in Figure 3 for a minimum of 7 h and up to 48 h. This range of time is appropriate for slightly to heavily melanized tissue. Although we notice condensation on the top of the lid, we have not noticed any tissue damage generated by heat when the light is placed at this distance.

**Pause point:** Samples may be transferred to 100% methanol and stored for up to six months at −20°C. As before, long-term storage should be done in microcentrifuge tubes (to prevent evaporation) in flammable safe freezers.

28. Rehydrate samples by transferring the basket through 500 μL each of decreasing MeOH/PBST solution: 75% methanol, 50% methanol, 25% methanol, and then switch to 100% PBST. Each step should be done on ice on a nutator at 25 rpm for 10 min.

**Figure 3:**
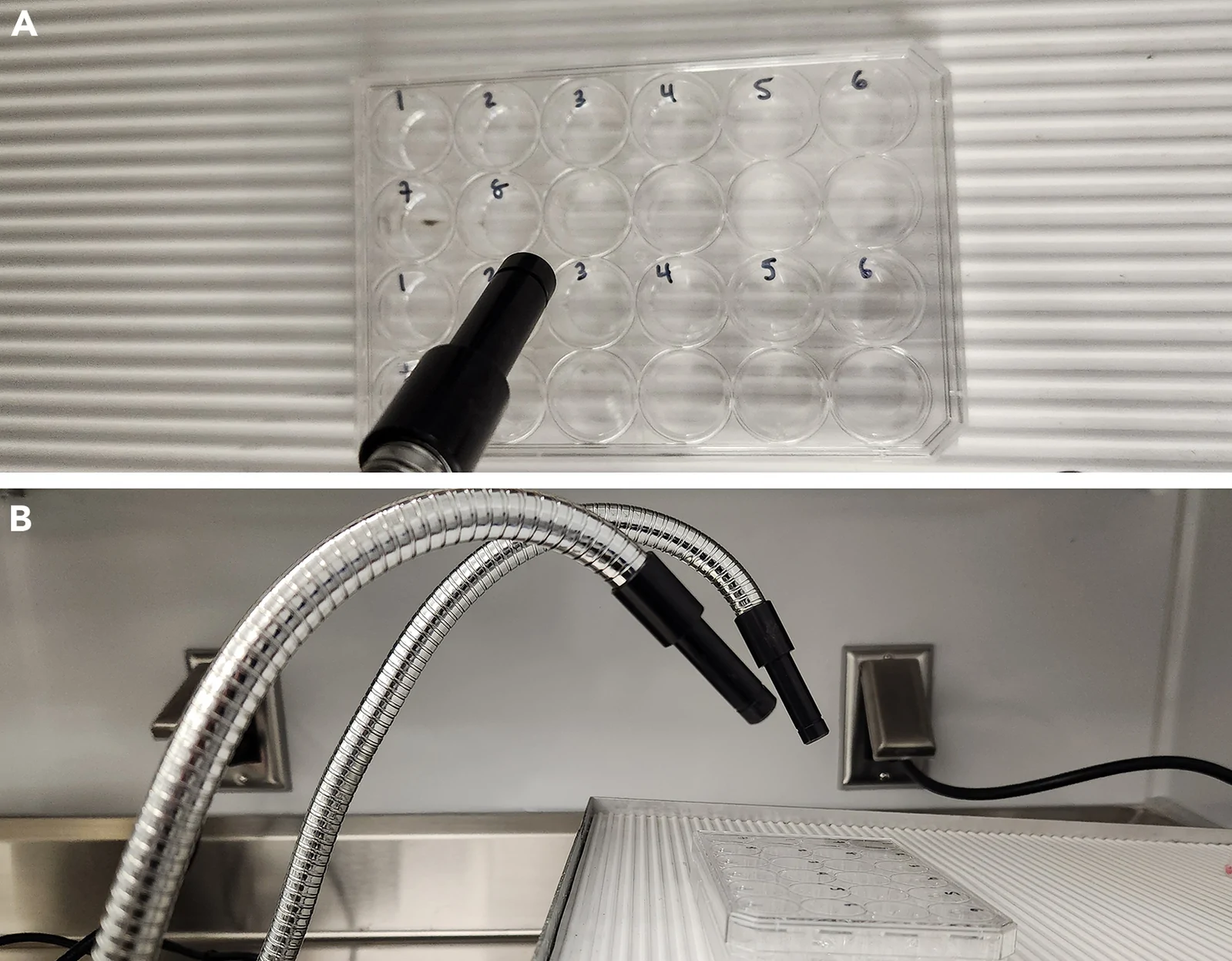
Bleaching setup to remove dark chromophores from the cuticle of insects (A) Setup in the cold room consisting of a white-light lamp and a 24-well plate on a nutator. (B) A side view showing the position of the plate relative to the light source.

***Note:*** Use sterile forceps to gently lift the basket from each solution and transfer it to the next. Excess liquid can be wiped off at the edge of the well before transfer. If the basket does not immediately submerge in the new solution, hold it at a 45°angle and place one end into the solution for 10 s before gently lowering the rest of the basket.

### Hybridization

**Timing: ∼1 h preparation + 16 h incubation**

**CRITICAL:** All materials should be disposed of in accordance with institutional safety and waste disposal policies. Care should be taken when handling methanol and reagents from Molecular Instruments, which contain formamide and other toxic chemicals. These reagents should be handled with caution according to your university and lab health and safety protocols. In our laboratory, all steps involving formamide-containing buffers are carried out in a certified chemical fume hood, and we recommend this practice.

29. Add 500 μL hybridization buffer to one well per sample on a new 24-well plate and allow it to pre-warm to 37°C for 5 min on a thermomixer.

***Note:*** This step is to acclimate the samples to the solvent change needed for hybridization. We have not found that the trace amounts of methanol in the mesh of the basket affect this pre-hybridization step. To save time, prepare one well for this pre-hybridization step and an additional well for the next step, the hybridization with probes.

30. Prepare the hybridization mix: 4 μL of 1 μM probe + 396 μL hybridization buffer (10 nM final).
31. Place the 24-well plate with the lid on top of a thermomixer with the microcentrifuge or PCR tube top removed so that the well is in contact with the heat block.

***Note:*** Any form of shaking incubator that can maintain 37°C temperature with shaking from 100–300 rpm is suitable.

***Note:*** The 300 rpm is the highest we use for our samples. If you find your tissue is sensitive to damage at these speeds, decrease accordingly, but maintain some motion to allow distribution of probes during hybridization.

32. Incubate samples at 37°C and 300 rpm overnight (16 h).

### Amplification

**Timing: ∼2 to 3 h preparation + 16 h incubation**

33. Prepare a heat block or thermocycler set to 95°C for denaturation of the hairpins. Following heating, the hairpins will be cooled to room temperature as described below.

***Note:*** Any heat block that will heat PCR tubes to 95°C, even more complicated setups like thermocyclers or thermomixers, will work. No motion or additional temperature change is required. If these are unavailable, holding the samples in a water bath with tweezers may work, though we have not tried it.

***Note:*** Turning on the heat block at this stage allows it to be heated to 95°C by Step 39. If only one heat block is available, this can be done between Steps 37 and 38, when the samples will be incubated at RT.

34. Add 500 μL probe wash buffer to four new wells per sample and allow it to preheat to 37°C.
35. Wash hybridized samples 4 × 15 min in the 500 μL probe wash buffer at 37°C by transferring baskets to new wells each time.
36. For each sample, prepare three new wells: add 500 μL of 5× SSCT to each of two wells and 500 μL of amplification buffer to the third well.

***Note:*** During Step 36, let samples cool to room temperature.

37. Wash samples 2 × 5 min in the 500 μL 5× SSCT at RT by transferring the baskets to the new wells.

**Pause point:** Samples may be stored at RT in SSCT for a maximum of 24 hours until the amplification step is performed. We recommend, however, that amplification be done on the same day.

38. Incubate samples for at least 5 min in the 500 μL amplification buffer (pre-amplification) from Step 36, or until hairpins are ready.
39. Prepare hairpins:
  a. Pipette 6 μL of each hairpin (h1 and h2) per sample into individual PCR tubes.
  b. Make sure that the hairpins are separated during this step into individual tubes and are only combined in Step 42.
  c. Denature the hairpins at 95°C in the PCR tubes for 90 seconds.
  d. Cool the hairpins at RT for 30 min in the dark (e.g., a cabinet).
40. For each sample, transfer the amplification buffer from the corresponding well of the 24-well plate into an individual PCR tube.
  a. Using a pipette with a tip opening appropriate for the specimen, aspirate the sample directly from the basket in a small volume of the same amplification buffer.
  b. Then transfer it to the corresponding PCR tube.

***Note:*** For larger specimens, the pipette tip can be cut to create a wider opening.

41. Subsequently remove all but a small amount of the buffer with a pipette.
42. Add 100 μL amplification buffer + 12 μL hairpins (6 μL each of h1 and h2) + 0.3 μL DAPI to each PCR tube.

***Note:*** DAPI is added here for convenience but can be added at any step after this at a 1:300–1:500 dilution for at least 1 h. At these dilutions, we do not observe overstaining or increased background after overnight incubation.

43. Incubate overnight (minimum of 12 h and maximum of 16 h) in the dark (e.g., covered in foil) at RT on a shaker set to 300 rpm.

***Note:*** As with the hybridization step, the speed can be decreased, but some shaking must be maintained to allow the amplifiers to diffuse in the solvent.

***Note:*** Incubation with amplifiers past 16 h may lead to increased non-specific binding as evidenced by signal in off-target regions such as cuticle or extracellular space.

44. To stop the amplification reaction, transfer the samples from the PCR tubes to baskets in wells containing 500 μL 5× SSCT.

***Note:*** The same baskets used the previous day can be reused.

***Note:*** At this stage, the amplifiers and probes are bound to the target RNA, and the sample no longer require stringent RNase-free conditions.

45. Wash samples 2 × 5 min and 3 × 30 min with 500 μL 5× SSCT in the dark using the basket and 24-well plate system.

***Note:*** If amplifiers appear to bind nonspecifically (e.g. broad punctate signal in cuticular and extracellular space that differs in pattern across many samples in the same stain), the final wash steps can be extended to 1 hour each.

### Immunohistochemistry

**Timing: Variable**

The second half of this protocol describes immunohistochemistry (IHC). If IHC is performed directly after FISH on the same samples, continue from the final 5× SSCT wash (Step 45) and transfer the samples into a 1:1 mixture of blocking buffer and 5× SSCT before proceeding with the steps below.

For IHC following FISH, we recommend keeping the samples in the same baskets and 24-well plate used for FISH. If antibody availability is limited or a high antibody concentration is required, samples can instead be transferred to microcentrifuge or PCR tubes to permit primary and secondary antibody incubations in volumes of 100 μL or less. Because the fluorophore-conjugated HCR amplifiers are already bound to the sample after FISH, all subsequent IHC steps should be performed under low-light conditions, with incubations kept completely in the dark (e.g., by wrapping the plate or tubes in aluminum foil).

We recommend performing FISH before IHC so that RNase-free conditions can be maintained through HCR amplification, after which IHC can be performed under standard clean laboratory conditions. Although we have not tested the reverse order, we expect that IHC followed by FISH may also be compatible if required.

If IHC is performed without preceding FISH, begin after rehydration from 100% methanol in Step 28. In this case, steps may be performed in the light until fluorophore-conjugated reagents are introduced, such as during the secondary antibody incubation. IHC alone can be performed using the basket and 24-well plate setup or, because it involves substantially fewer solution changes and wash steps, entirely in microcentrifuge tubes.

Incubation times should be optimized for the antibody and specimen. In our experience, primary antibody incubation times range from 1 to 10 days depending on antibody performance and tissue thickness.

46. Wash 3× 5min in PBST (1% Triton X-100).

***Note:*** The PBST used in IHC is with Triton X-100 instead of Tween©-20.

47. Incubate samples in Normal Donkey Serum blocking buffer (NDS-BB) with 0.5% Triton X for 1 h at RT
48. Dilute primary antibodies in NDS-BB with 0.5% Triton X. We have used dilutions ranging from 1:100 to 1:10,000 depending on the antibody.

***Note:*** Testing several dilutions may be required before final experiments. We recommend doing this separately before coupling the experiment with FISH. To start, we recommend a primary incubation time of at least 2 days. The proper dilution is the minimum amount of antibody that gives a clear signal using reasonable imaging parameters. Once the proper dilution is obtained, the incubation period can be adjusted (decreased without sacrificing signal or increased to improve signal).

49. Incubate in primary antibody at RT for the incubation period necessary for your antibody (usually 1 to 10 days for *O. biroi* antennae).
50. Wash 3× 5 min in PBST (1% Triton X-100).
51. Incubate in secondary antibody in NDS-BB with 0.5% Triton X for 1 to 2 days at RT.

***Note:*** Additional nuclear or cytoskeleton stains can be added at this stage for convenience. If these stains are strong enough, they can alternatively be added via a short incubation period in the subsequent step.

***Note:*** Incubation time for secondary antibody may also vary, but 1–2 days at RT is a reasonable starting point.

52. Wash 3× 5 min in PBST (1% Triton X-100).

### Imaging

**Timing: ∼1 to 2 h for preparation; variable for imaging**

53. Prepare frosted slides for mounting by using a Kimwipe on both sides of the slide with 95% EtOH.
54. Label with sample number, conditions (e.g., sonicated, bleached), and date on the frosted section. Place Avery® Self-Adhesive Hole Reinforcement Binder Stickers (see Figure 4 for example) onto the surface of the slide to create a well for the samples.

**Figure 4:**
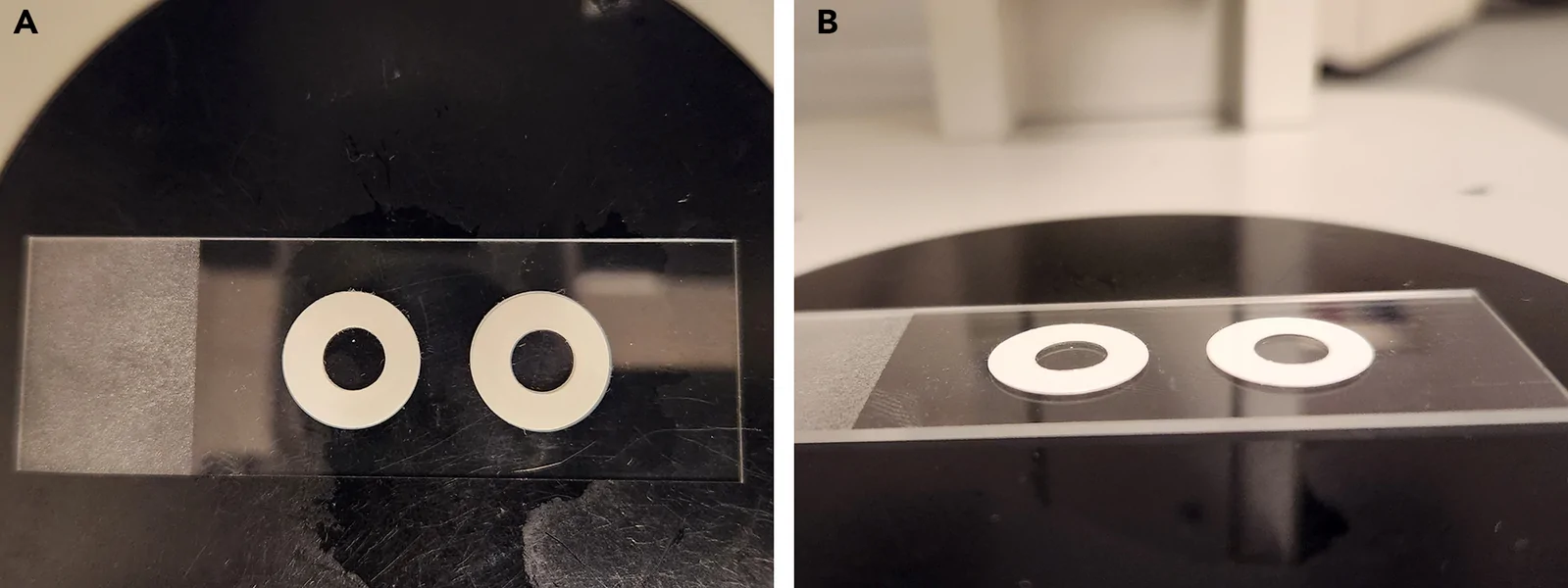
Setup for mounting antennae using binder rings to create a well on slides (A) Top view of slides with two binder ring wells each made up of two binder rings placed on top of each other. (B) Side view of A.

***Note:*** We recommend 2 binder stickers per well per slide, but thicker samples may require more binder stickers. Also, we recommend no more than 2 wells per slide because if a slide is too densely populated, mounting and imaging may become difficult.

55. Using a pipette appropriate for the size of your samples, transfer 50 μL PBST containing samples to the binder wells and remove excess liquid with a pipette.

***Note:*** Remove as much PBST as possible without allowing the tissue to dry. We do not rinse in mounting medium before this step because small, transparent tissue samples become difficult to relocate once in the mounting medium. The residual PBST volume is small enough that it does not interfere with mounting.

56. Add 8 μL of SlowFade™ Glass Antifade mountant to the well and orient tissue using an eyelash tool (a human eyelash superglued to the end of a pencil or thin stick).

***Note:*** Any instrument that allows for manipulation of the tissue without damaging it can be used to arrange the tissue in the preferred orientation.

***Note:*** We have found that SlowFade™ Glass Antifade mountant and SlowFade™ Diamond Antifade mountant work well with this protocol, though we obtain better clearing with SlowFade™ Glass Antifade mountant as it has a higher refractive index. We recommend imaging within a week after mounting in SlowFade™ Glass Antifade mountant as we observe a decrease in signal after that.

57. Seal the coverslip with nail polish. Let nail polish airdry in a low humidity, dark area (e.g., a cabinet) for 30 min.

**CRITICAL:** Avoid air bubbles and over-compression during mounting.

**Pause point:** Place the slides in a container where the individual slides are separated and store at 4°C. While we recommend imaging soon after mounting for best results, we have not noticed significant loss of signal if imaged within a week. When ready to image, take samples out from 4°C and let them warm to room temperature before imaging to prevent warping or excessive condensation.

***Note:*** Images were acquired with a Zeiss LSM 900 confocal microscope using 405 nm, 488 nm, 561 nm, and 640 nm lasers. Most images were acquired using a Zeiss LD LCI Plan-Apochromat 40×/1.2 NA multi-immersion objective immersed in Zeiss Immersol-G. A 20×/0.8 NA Plan-Apochromat objective was used for a subset of overview acquisitions. Laser power was adjusted individually for each fluorophore to avoid underexposure or saturation and was typically set to 0.2–5%, and up to 10% for some 405-nm acquisitions. The pinhole was generally set to approximately 1 Airy unit, detector gain was typically 500–680 V, digital gain was 1, and no detector offset was applied. Images were acquired in integration mode, generally using two-fold signal averaging and bidirectional scanning, with a typical pixel dwell time of approximately 0.38 μs. A small subset of acquisitions used single averaging, unidirectional scanning, or longer dwell times.

***Note:*** Z-stacks were acquired at frame sizes ranging from 992 × 987 to approximately 2048 × 2052 pixels, with lateral sampling ranging from approximately 0.078 to 0.693 μm/pixel and z-step sizes ranging from 0.60 to 2.00 μm. High-resolution acquisitions were generally collected at approximately 0.078–0.097 μm/pixel, providing approximately Nyquist or better lateral sampling for the principal fluorescence channels used with the 40×/1.2 NA objective. Lower-resolution acquisitions, including images acquired at 992 × 987 pixels, were intended primarily for larger-field overview imaging and were not acquired at Nyquist sampling. Choice of objective, frame size, lateral sampling, and z-step size reflected the spatial scale and purpose of each acquisition, as well as acquisition-time and microscope availability constraints. Smaller z-steps were used when greater axial detail was required, whereas larger z-steps were used for thicker specimens or overview stacks to reduce acquisition time and photobleaching. Detection ranges were adjusted according to fluorophore and included 400–560 nm, 400–580 nm, 400–585 nm, 400–605 nm, 410–605 nm, 560–700 nm, 636–700 nm, and 645–700 nm.

***Note:*** We recommend starting with the imaging parameters usually used in your laboratory and adjusting resolution, step sizes, and laser power as needed.

## Expected outcomes

This protocol enables whole-mount staining of cuticular and melanized tissues, particularly insect antennae. Starting from an intact antenna, the combined use of sonication and bleaching allows visualization of protein and mRNA throughout the entire volume. This approach eliminates the need for cryosectioning, which is low throughput, can distort the morphology of small tissues, and typically permits analysis of only a limited subset of the total volume.

The sonication and bleaching setups are simple and support high throughput processing of several dozen antennae simultaneously with minimal hands-on time (Figures 2 and 3). Bleaching effectively removes melanin and substantially improves imaging quality, particularly for short wavelength fluorophores (Figures 5 and 6). Gentle sonication is effective in permeabilizing whole tissues, allowing robust probe and antibody penetration through the cuticle (Figure 7). Putting everything together, the combination of custom designed probes, sonication, and bleaching allows us to visualize olfactory receptor expression in the ant antenna (Figure 8). Although the protocol was optimized for *O. biroi* antennae, it can be readily adapted to other ant tissues of similar composition, such as thorax and legs (Figure 9). Furthermore, it works equally well for other arthropod species, such as the red flour beetle *Tribolium castaneum* (Figures 10 and 11). Here, bleaching was extended to 48 hours due to the more extensively melanized *T. castaneum* cuticle (Figure 10).

**Figure 5:**
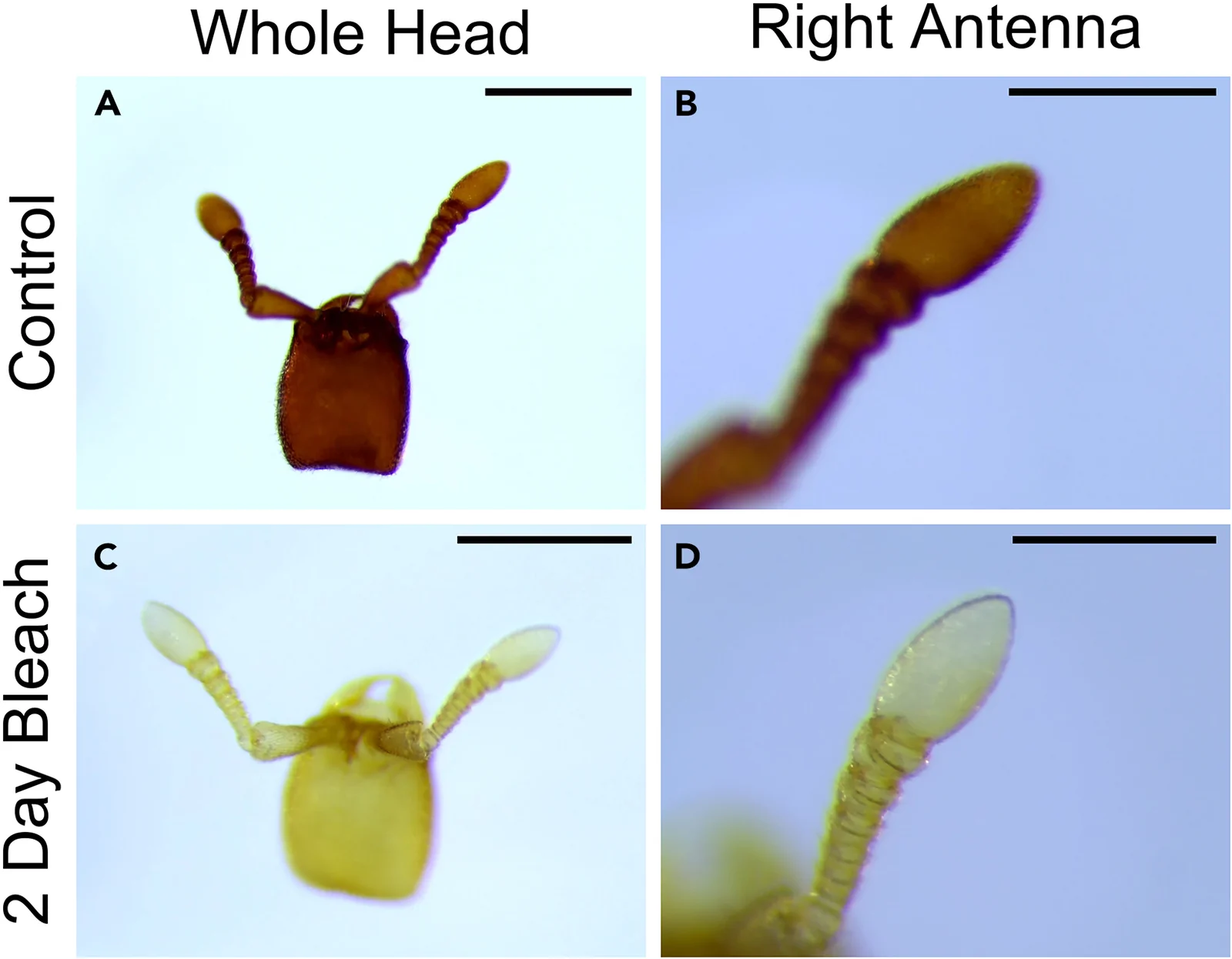
Bleaching lightens the cuticle of *Ooceraea biroi* antennae (A and B) Control *O. biroi* head and antenna not subjected to peroxide bleaching. (C and D) *O. biroi* head and antenna that was bleached with peroxide. (B and D) Magnifications of the right antenna in A and C, respectively. Ants were taken from the same stock colony. The control samples were placed in 100% methanol, while the experimental samples were subjected to 3% hydrogen peroxide in 90% methanol. The total experiment time was 2 days. Scale Bars: A, C: 500 μm; B, D: 250 μm.

**Figure 6:**
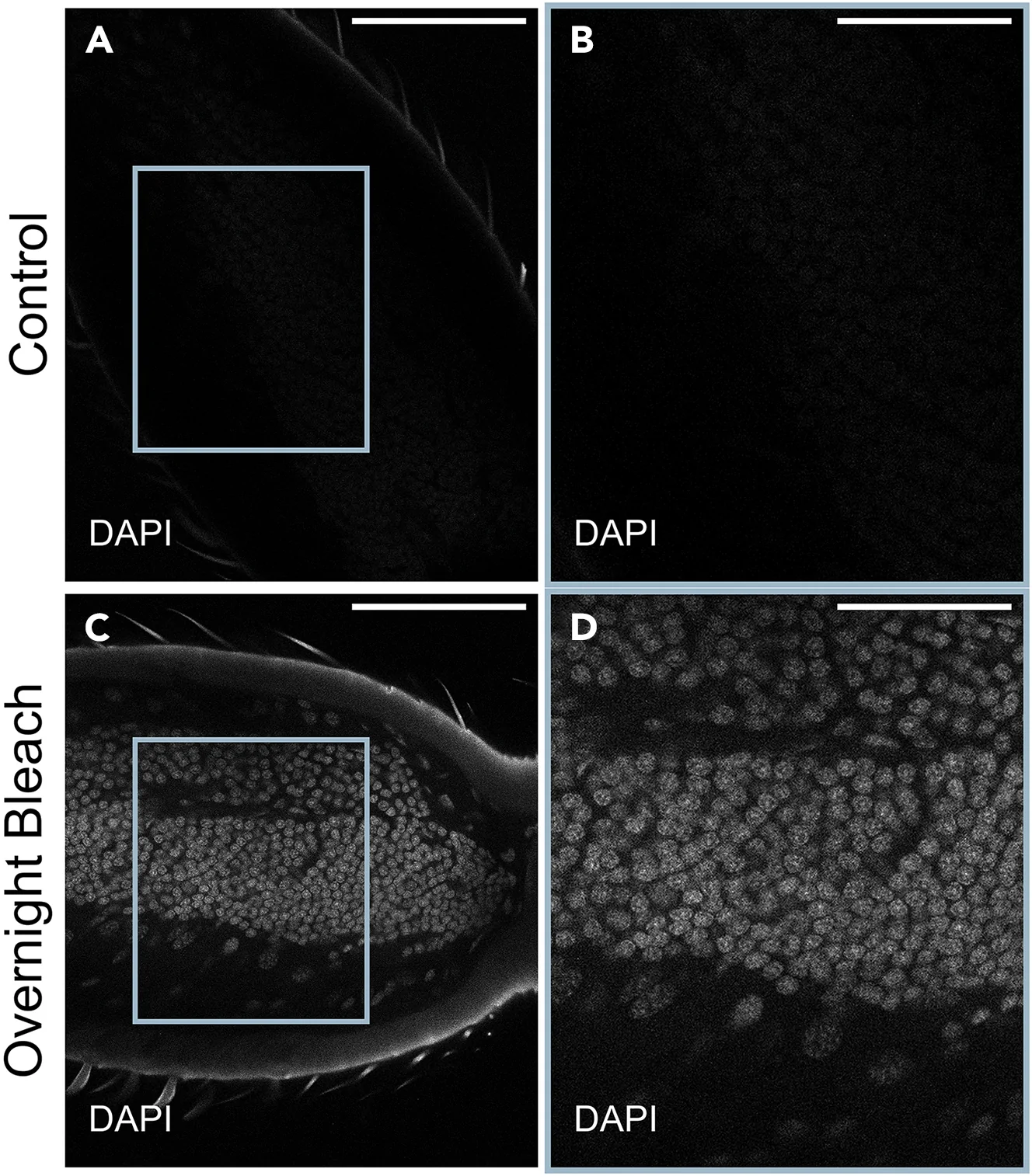
Bleaching enables visualization of nuclei/DNA through melanized cuticle in *O. biroi* antennae (A and B) Control samples. (C and D) Experimental samples bleached overnight. (B and D) Magnified views of the cyan boxes in panels A and C, respectively. Ants were taken from the same stock colony and their antennae dissected. The control antennae were placed in 100% methanol while the experimental antennae were subjected to 3% hydrogen peroxide in 90% methanol. Both samples were then incubated with DAPI at a dilution of 1:500 overnight. DAPI was detected only in the bleached samples. Images were acquired using identical laser power and gain settings. Scale Bars: A, C: 50 μm; B, D: 25 μm.

**Figure 7:**
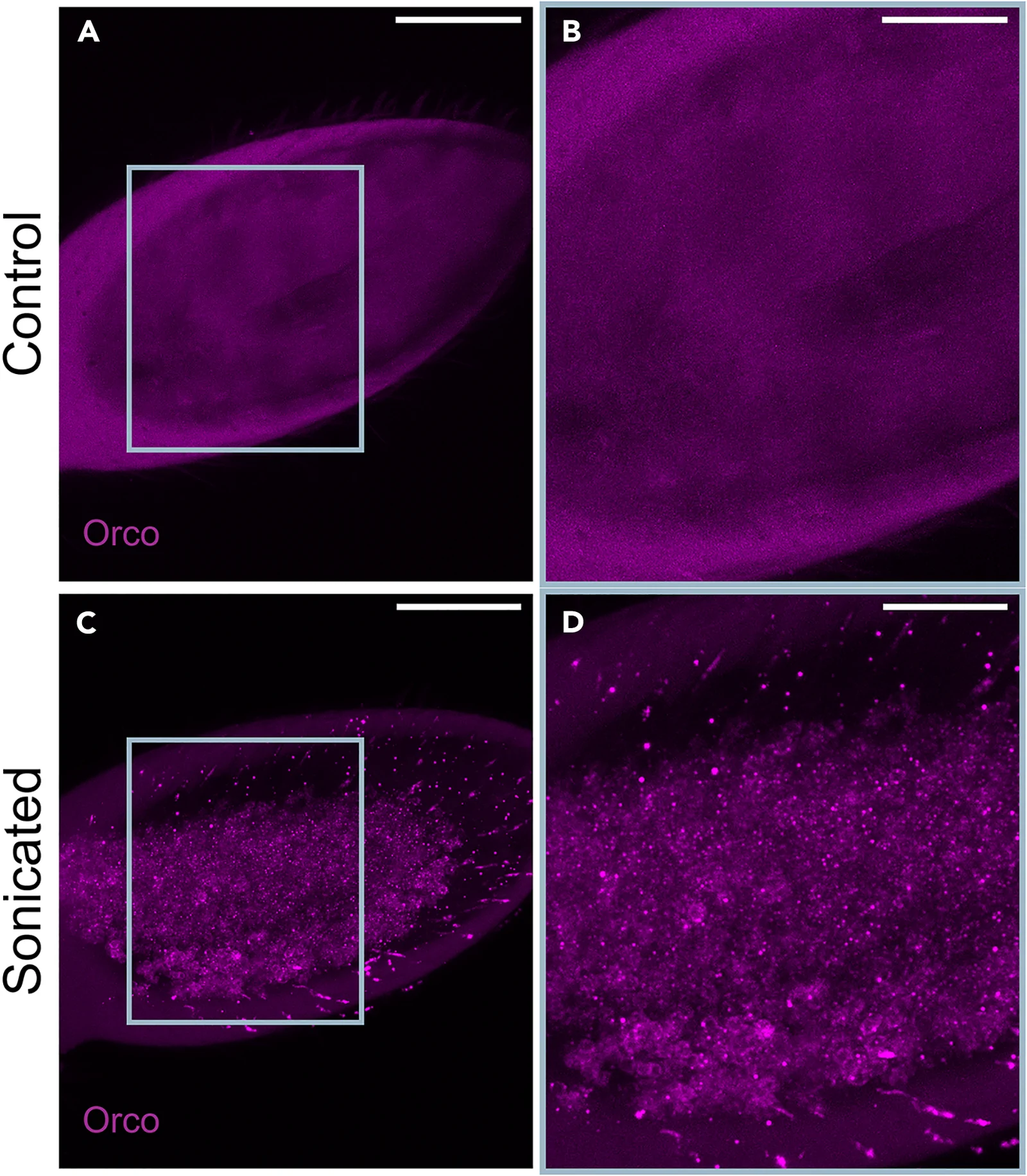
Sonication enables antibody penetration in unbleached *O. biroi* antennae (A and B) Control antenna dissected but not subjected to sonication. (C and D) Antenna sonicated using standard parameters of amplitude 10 with 2 s ON and 20 s OFF for 20 cycles. (B and D) Magnified views of the cyan boxes in panels A and C, respectively. The images represent stacks of multiple z-slices. Samples were stained with an Orco primary antibody (1:250) in NDS blocking buffer for 2 days and an Alexa Fluor 647 (1:500) secondary antibody in NDS blocking buffer for 1 day. Sonication enabled antibody penetration throughout the antennal tissue, and Orco staining is detectable only in the sonicated samples. Images were acquired using identical laser power and gain settings. Scale Bars: A, C: 50 μm; B, D: 25 μm.

**Figure 8:**
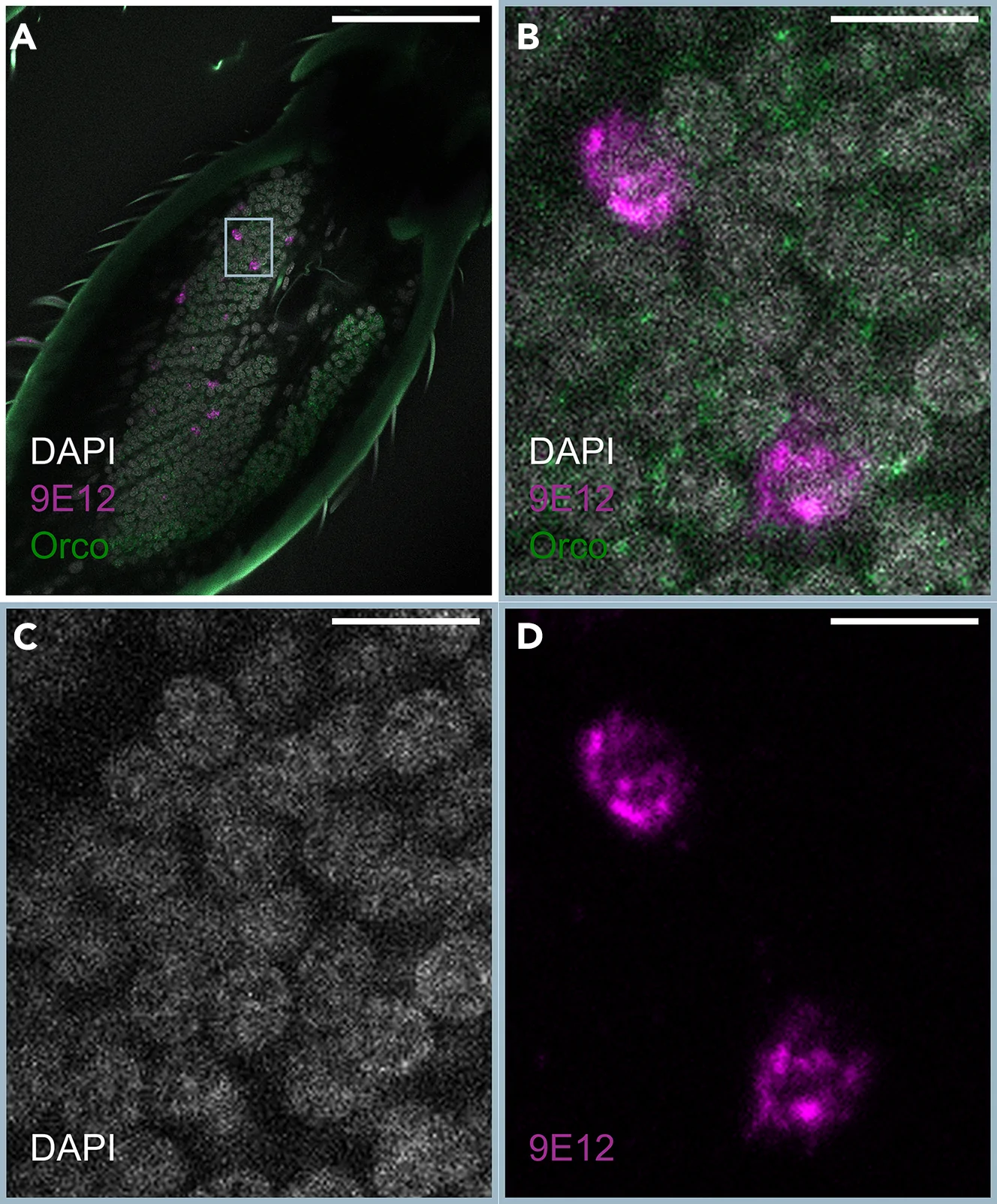
The combination of custom probe design, sonication, and bleaching allows for clear detection of odorant receptor (OR) RNA via RNA-FISH in a whole-mount adult *O. biroi* antenna (A) Composite image of DAPI, OR 9E12, and Orco RNA staining. (B–D) Magnified view of the cyan box in (A), with several Orco positive cells and two Orco + OR 9E12 positive cells. (C) and (D) show the separate DAPI and OR 9E12 channels, respectively. Orco and OR 9E12 were detected with 488 and 647 fluorophores, respectively. Scale Bars: A: 50 μm; B, C, D: 5 μm.

**Figure 9:**
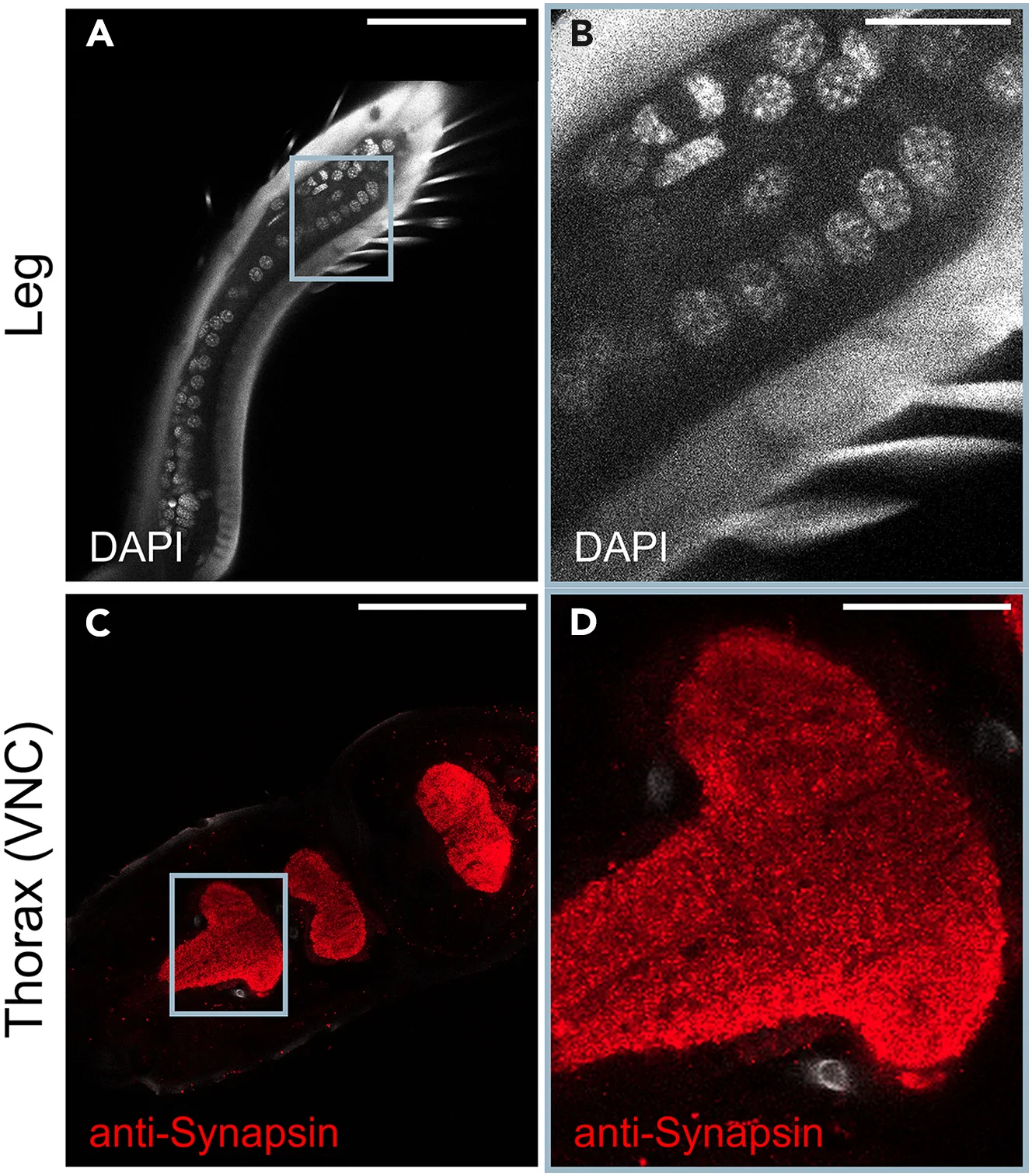
Sonication and bleaching are compatible with *O. biroi* leg and thorax staining (A and B) *O. biroi* leg stained for DAPI (1:500 for 1 hour). (C and D) *O. biroi* thorax and ventral nerve cord (VNC) stained with anti-Synapsin (1:20) for 4 days, followed by Alexa Fluor 647–conjugated donkey anti-mouse IgG (1:250) and DAPI (1:500) for 2 days. (B and D) Magnified views of the cyan boxes in panels A and C, respectively. All samples were fixed for 2 h before sonication, sonicated, fixed for an additional 2 h after sonication, and bleached overnight. Scale Bars: C: 200 μm; A, D: 50 μm; B: 10 μm.

**Figure 10:**
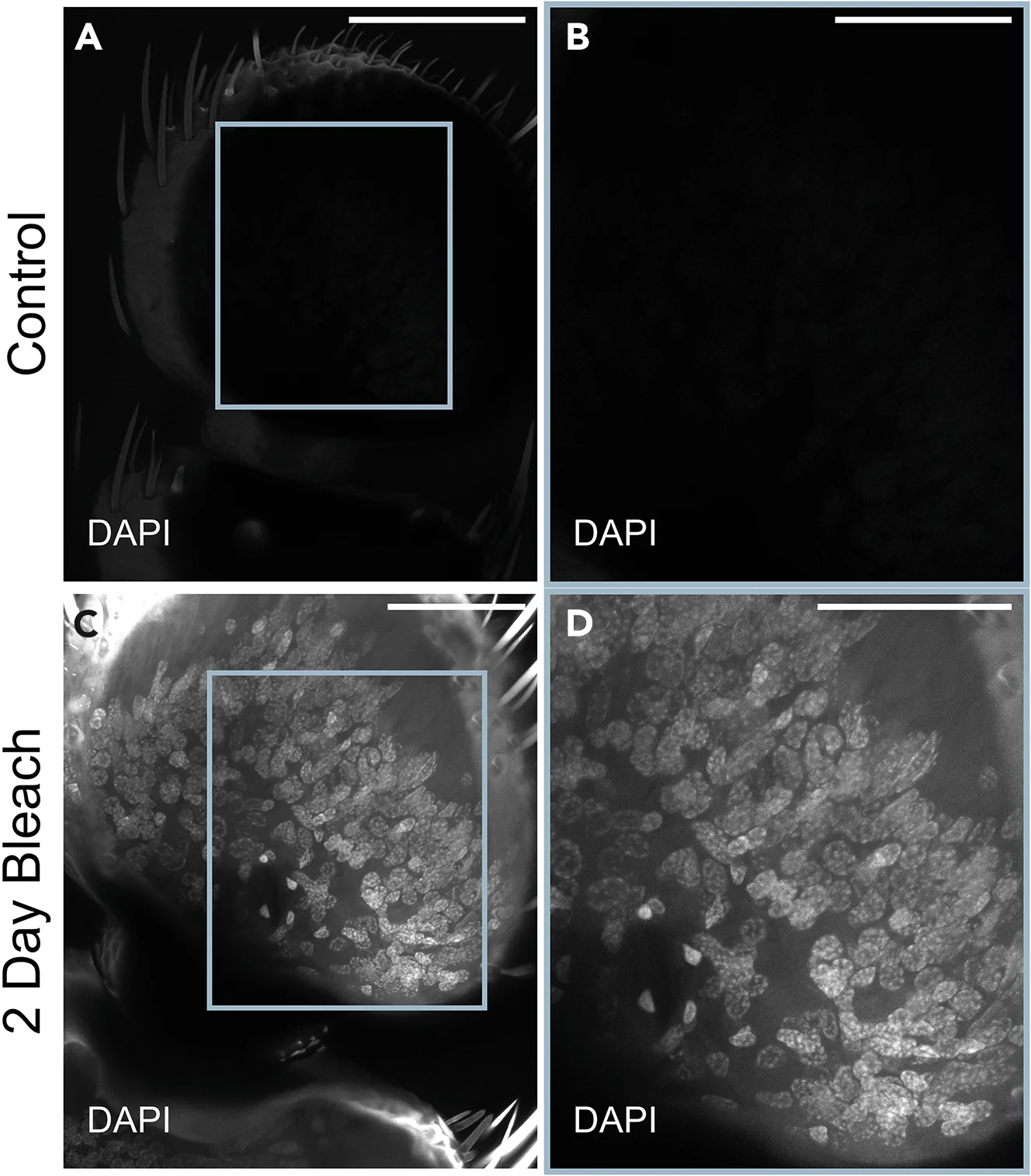
Bleaching is required for nuclear/DNA staining in *T*. *castaneum* antennae (A and B) Control samples. (C and D) Samples bleached for 48 hours. (B and D) Magnified views of the cyan boxes in panels A and C, respectively. All samples were sonicated and incubated with DAPI at a dilution of 1:500. Nuclear signal was detected only in the bleached samples. Images were acquired using identical laser power and gain settings. Bleaching for two days and overnight incubation in 1:500 DAPI was required to obtain robust signal. Scale Bars: A: 50 μm; C: 30 μm; B, D: 25 μm.

**Figure 11:**
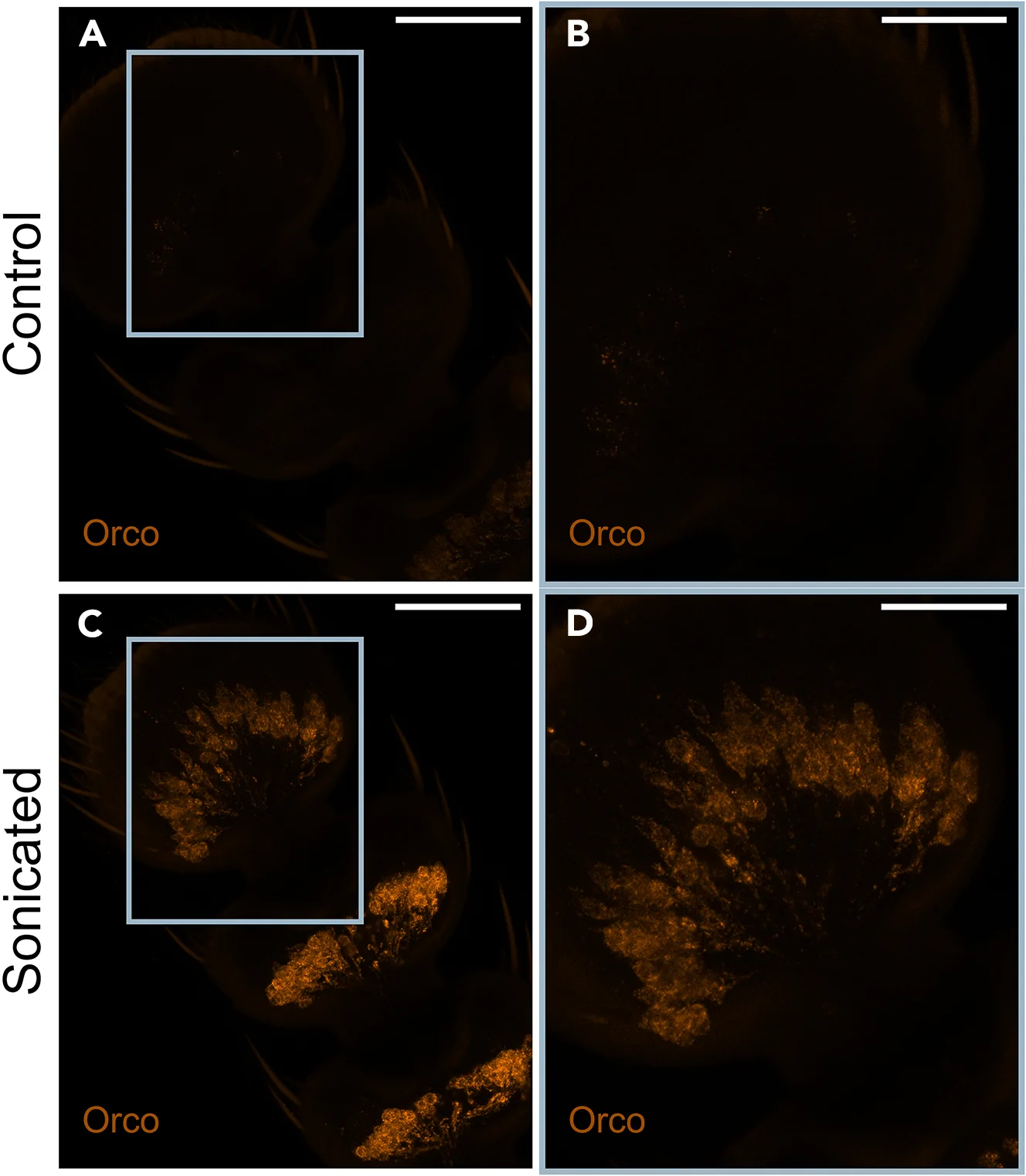
Sonication enables RNA probe penetration in unbleached *T. castaneum* antennae (A and B) Control samples not subjected to sonication. (C and D) Sonicated samples. (B and D) Magnified views of the cyan boxes in panels A and C, respectively. The images represent stacks of multiple z-slices. The three distal antennal segments are shown. Orco RNA-FISH was performed on these samples. Signal was amplified using a fluorophore detected at 647 nm. Scale Bars: A, C: 50 μm; B, D: 25 μm.

## Limitations

While we find the sonicator more effective than enzymatic digestion for thick cuticle such as those of ants and beetles, we realize that not all labs may have access to a sonicator. However, sonicators are frequently used for protein extraction and DNA shearing, and they are common equipment in molecular biology departments. We therefore hope that many researchers will have initial access to a sonicator to try our protocol on their respective samples. If the protocol proves useful for their tissue of interest, purchasing a sonicator may be worth it in the long run because anyone can use it without training, and no additional materials are needed. Purchasing a sonicator might be advantageous compared to purchasing a microtome, because it does not require training someone to cut small tissue and it produces whole-mount volumetric data. A heat block that can reach 37°C with shaking is also required, but there are many options, from hybridization ovens to shaking incubators that a lab can use or borrow. The only step requiring high temperature is the 95°C incubation to prepare the separate amplifier hairpins, though this is only for 90 seconds and a water bath may be adapted for this step. All other required materials are readily available, nonhazardous, and easily integrated into existing staining workflows in most laboratories.

This protocol has been optimized for tissue from late pupal (the last 11 of ∼17 days of metamorphosis) to adult stages of *O. biroi*. Tissue from early pupae (the first 6 of ∼17 days of metamorphosis), which are softer and less melanized, often disintegrate during the sonication step and do not benefit from bleaching. In practice, staining early pupal tissue using this protocol is frequently more challenging than staining adult tissue.

The sonication amplitudes and cycle parameters described here do not appear to disrupt tissue integrity or degrade RNA or protein, based on the staining outcomes observed. However, these parameters may not be universally applicable to all tissues or experimental conditions, and optimization may be required for other sample types.

Bleaching introduces additional limitations. While nuclear stains such as DAPI remain effective following bleaching, some stains, such as phalloidin, are not compatible with the bleaching step.

So far, we have applied this protocol in combination with RNA-FISH only in antennal tissues of *O. biroi* and *T. castaneum*. The examples of other tissues provided here involve nuclear and antibody staining (Figure 9). However, we predict that the RNA-FISH protocol described here would be equally efficient for staining and imaging other arthropod body parts.

## Troubleshooting

### Problem 1

Tissue appears necrotic or degraded after imaging.

### Potential solution

This is often caused by prolonged dissection time at room temperature. Antennae should be placed into cold formaldehyde immediately after dissection if done at room temperature. To improve speed and tissue preservation, dissections can be performed on top of a large dish filled with ice or dry ice so that tissues and solutions remain cold throughout the procedure. Dissect antennae in small batches and transfer them promptly into cold fixative. If necrosis persists, ensure that the formaldehyde solution is fresh and has not been stored for extended periods. Improper fixation, including under-fixation, can also affect tissue integrity.

### Problem 2

High background or diffuse autofluorescence across imaging channels.

### Potential solution

High background during imaging can be caused by over-fixation. Over-fixed tissue exhibits hazy background fluorescence and reduced contrast between signal and background. Reduce fixation time or fixative concentration and confirm that fixation conditions match those described in the protocol.

### Problem 3

Tissue is destroyed or fragmented after sonication.

### Potential solution

This indicates over-sonication, which can occur if the amplitude is too high or if the tissue is too soft. Over-sonicated samples often result in a slurry or mush. Reduce the sonication amplitude.

### Problem 4

Little or no improvement in probe or antibody penetration after sonication.

### Potential solution

This may result from under-sonication or failure of the sonicator to initiate properly. Confirm that the sonicator is functioning and that energy is being delivered. Even with hearing protection you can faintly hear the sounds of sonication which indicate that the sonicator is working. We have observed effective permeabilization at amplitudes as low as three.

### Problem 5

Bubbles form within the tissue during the peroxide methanol bleaching step.

### Potential solution

Bubble formation indicates incomplete dehydration and the presence of residual water reacting with peroxide. If this occurs, bubbles have a high chance of forming near the tissue. Bubbles can enter the tissue through openings, and the increased pressure in cuticle encased tissue can lead to deformations. We therefore recommend discarding the affected tissue unless the sample represents tissue in limited supply. Repeat dehydration using fresh samples and extend dehydration steps from 10 to 20 min. Alternatively, samples may be left in 100% methanol overnight before proceeding to bleach the following day.

### Problem 6

Inconsistent or incomplete bleaching across samples.

### Potential solution

Variation in bleaching efficacy may reflect biological differences in melanin content. In addition, dense packing of tissues within a centrifuge tube can lead to uneven light exposure. To improve consistency, reduce the number of samples per tube (if tubes are used) or use a wider container that allows tissues to lie in a single layer, ensuring uniform exposure to light and peroxide. Using a rocker might also allow even exposure, resulting in improved bleaching. Additionally, in some cases, longer incubation may be required for proper bleaching. We have not noticed any adverse effects of bleaching for up to 3 days on tissue integrity or FISH/IHC signal. Bleaching beyond 3 days with successful staining may still be possible, but we have not attempted it. For example, *T. castaneum* requires longer incubation (at least approximately 48 hours) than *O. biroi* (at least approximately 7 hours) due to the darker initial coloration. Testing the incubation time can be done qualitatively, by observing the change in color under a stereoscope over several hours or days, or by DAPI staining and imaging.

### Problem 7

Samples dry out during overnight hybridization.

### Potential solution

When using baskets in multi-well plates, ensure that at least 350 μL hybridization buffer is used per well for a standard 24-well plate. Smaller volumes increase the risk of complete evaporation and tissue desiccation during overnight incubation.

### Problem 8

Certain stains fail after bleaching.

### Potential solution

Some stains, such as phalloidin, are not compatible with the bleaching step even when applied after bleaching. All stains should be empirically tested. To date, we have not observed issues with Alexa Fluor dyes, Molecular Instruments amplifier fluorophores, or DAPI.

### Problem 9

HCR does not produce a signal or produces too much background.

### Potential solution

In our experience, the probes from Molecular Instruments or ProbePy do not fail often (close to 100% success on first staining). We find that when we fail to see signal on the first attempt, we usually see it on the second attempt, suggesting human error in the execution of the RNA-FISH protocol. If your probes consistently fail, we recommend trying the protocol with another set of probes as there might be some secondary structure in the target RNA or an RNA-binding protein inhibiting the binding of the first set of probes. Additionally, after mounting in SlowFade™ Glass mounting medium, we notice a decrease in signal after one week and have not imaged past two weeks. Other mounting media may preserve the signal for longer, but we recommend imaging soon after mounting for best results. If there is too much background (i.e., diffuse, non-specific puncta in cuticle or extracellular space), we recommend decreasing the amplifier incubation time (but not to less than 12 hours) or fixation time, because both can affect non-specific binding. Additionally, we find that some tissues (e.g., chemosensory sensilla and fat tissue) often display broad non-specific binding across many probes for unknown reasons.

## Resource availability

### Lead contact

Any further information about the article and requests should be directed to the lead contact, Daniel J.C. Kronauer (dkronauer@rockefeller.edu).

### Technical contact

Any technical questions on executing this protocol should be directed to the technical contact, P. Daniel H. Pastor (phejazi@rockefeller.edu), and specific questions about ProbePy should be directed to Giacomo L. Glotzer (gglotzer@rockefeller.edu).

### Materials availability

Any requests for HCR probe sets used in this study should be directed to Daniel J.C. Kronauer (dkronauer@rockefeller.edu).

### Data and code availability

All code used for probe design is on GitHub with links provided in the key resources table.

## Acknowledgments

We thank Stephany Valdés-Rodríguez for ant maintenance and providing experimental animals and Leonora Olivos-Cisneros for valuable input on RNA-FISH and tissue sonication methods. The RNA-FISH protocol was adapted from a method originally developed by Margo Herre and subsequently modified by Leah Houri-Zeevi in Leslie Vosshall’s laboratory.^8^ We also thank Eliza Jaeger for her advice on bleaching ant cuticles based on protocols she helped develop. The anti-Synapsin antibody was developed by Erich Buchner and obtained from the Developmental Studies Hybridoma Bank, created by the National Institute of Child Health and Human Development of the National Institutes of Health and maintained at the University of Iowa, Department of Biology. This work was supported by the 10.13039/100000002National Institutes of Health via the 10.13039/100000065National Institute of Neurological Disorders and Stroke under award no. R01NS123899 (D.J.C.K.), NRSA Training Grant #GM144299 (G.L.G.), and a Medical Scientist Training Program grant from the 10.13039/100000057National Institute of General Medical Sciences under award no. T32GM152349 to the Weill Cornell/Rockefeller/Sloan Kettering Tri-Institutional MD-PhD Program (P.D.H.P.). The content is solely the responsibility of the authors and does not necessarily represent the official views of the National Institutes of Health. This work was also supported by the 10.13039/100000011Howard Hughes Medical Institute, where D.J.C.K. is an investigator.

## Author contributions

P.D.H.P. devised and performed troubleshooting for sonication, conducted RNA-FISH and immunohistochemistry experiments, and assembled the figures. P.D.H.P. and G.L.G. performed troubleshooting related to the bleaching experiments. D.D.F. and G.L.G. contributed additional method improvements and input on the RNA-FISH, sonication, and bleaching procedures. A.H.-G. performed experiments for Figure 4. I.L. performed experiments for Figure 9. P.D.H.P. and D.J.C.K. wrote the manuscript, and all authors reviewed and approved the final version. D.J.C.K. supervised the project.

## Declaration of interests

The authors declare no competing interests.

## Supplemental information

Table S1. Probes for OR 9E12 (*Ooceraea biroi*)

Table S2. Probes for Orco (*Tribolium castaneum*)
